# Boosting the immunotherapy response by nutritional interventions

**DOI:** 10.1172/JCI161483

**Published:** 2022-06-01

**Authors:** Laurence Zitvogel, Guido Kroemer

**Affiliations:** 1 Gustave Roussy Comprehensive Cancer Institute, Villejuif, France.; 2 Université Paris Saclay, Faculty of Medicine, Le Kremlin-Bicêtre, France.; 3 INSERM U1015, ClinicObiome, Paris, France.; 4 Equipe Labellisée par la Ligue Contre le Cancer, Villejuif, France.; 5 Center of Clinical Investigations in Biotherapies of Cancer (CICBT) BIOTHERIS, Villejuif, France.; 6 Equipe Labellisée par la Ligue Contre le Cancer, Université de Paris Cité, Sorbonne Université, Institut Universitaire de France, Inserm U1138, Centre de Recherche des Cordeliers, Paris, France.; 7 Metabolomics and Cell Biology Platforms, Institut Gustave Roussy, Villejuif, France.; 8 Institut du Cancer Paris CARPEM, Department of Biology, Hôpital Européen Georges Pompidou, AP-HP, Paris, France.

## Introduction

**Figure 1:**
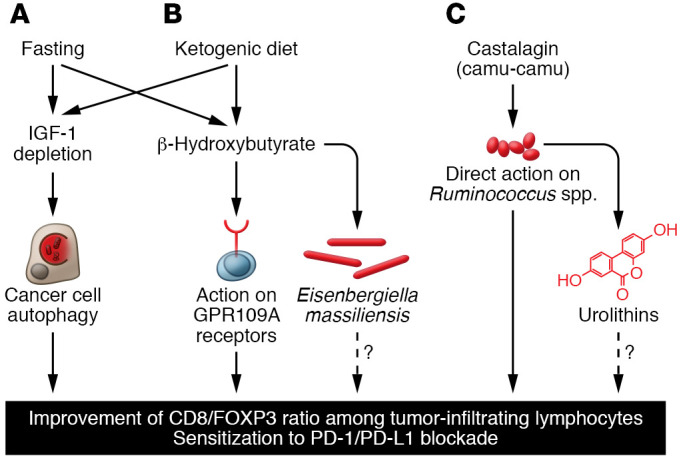
Dietary interventions for sensitizing to ICIs targeting PD-1/PD-L1. (**A**) Mechanisms of fasting-induced immunostimulation. Fasting causes a reduction of circulating IGF1 levels, which stimulates autophagy in cancer cells. (**B**) Ketogenic diet and its effects on the patient. Increased levels of the ketone body β-hydroxybutyrate stimulate GPR109A receptors expressed by immune cells and modify the gut microbiota (**C**) Supplementation with camu-camu–derived castalagin. In the intestinal lumen, castalagin directly acts on specific *Ruminococcus* species to stimulate their expansion. Some bacterial species, including *Ruminococcus* species, metabolize castalagin into urolithins, which might have immunostimulatory effects as well. All three dietary interventions modify the tumor microenvironment, hence enhancing the CD8/FOXP3 ratio in the immune infiltrate and sensitizing to immune checkpoint inhibition targeting the PD-1/PD-L1 interaction.

Immune checkpoint inhibitors (ICIs) targeting the interaction between programmed cell death protein 1 (PD-1) and programmed death-ligand 1 (PD-L1) constitute the backbone of the oncological armamentarium. Nonetheless, certain cancer types almost never respond to such ICIs, and only a minority (one-quarter to one-third) of patients bearing cancers for which PD-1 blockade is FDA approved obtain therapeutic benefit. For this reason, a fervent search for combination treatments, including other immunotherapies, immunogenic chemotherapies, or targeted therapies, is underway (1). Surprisingly, nutritional interventions are now being considered as a possible strategy for improving the efficacy of immunotherapy. Indeed, three rather different types of dietary interventions are being considered at the clinical level for boosting immunotherapeutic responses: (a) fasting regimens involving a reduction of caloric intake; (b) ketogenic diets, which are low-carbohydrate, high-fat, sufficient-protein diets leading to an increase in ketone bodies; and (c) the supplementation with polyphenol-rich extracts from an Amazonian berry, camu-camu (Figure 1).

## Fasting

Fasting, which can be either continuous (caloric restriction) or intermittent (through cycles that last 18 hours: time-restricted feeding; 1 day every second day: alternative-day fasting; 2 consecutive days per week: 5:2 day fasting, etc.), is well known to improve the health spans and life spans of laboratory animals (2). In obese patients, caloric restriction–induced weight loss is not further improved by time-restricted feeding (eating only between 8:00 am and 4:00 pm), suggesting that global caloric intake is the most important determinant of the metabolic consequence of fasting (3). However, when applied to mice, continuous caloric restriction appears to be more efficient in reducing the development and progression of cancer than intermittent fasting (4). Of note, a single fasting cycle of 48 hours administered together with immunogenic chemotherapeutics (such as anthracyclines or oxaliplatin) alone or together with PD-1 blockade improves tumor growth reduction (5–7). The mechanisms underlying the beneficial effects of fasting include the reduction in circulating IGF1 concentrations and the induction of autophagy in cancer cells (which favors the release of ATP for the recruitment of immune effectors into the tumor bed) as well as a favorable shift in the tumor microenvironment, with an increase in the ratio of CD8^+^ cytotoxic T lymphocytes over FOXP3^+^ regulatory T cells (refs. 5, 7, and Figure 1A).

These chemotherapy- and chemoimmunotherapy-enhancing effects are also obtained when fasting is replaced by the administration of “caloric restriction mimetics” such as hydroxycitrate, which also diminishes IGF1 levels, stimulates autophagy in malignant cells, and raises the CD8/FOXP3 ratio (5). Injection of recombinant IGF1 protein into mice abolishes all these hydroxycitrate effects, supporting the mechanistic importance of IGF1 reduction (5). Accordingly, pharmacological inhibition of IGF1 receptor (IGF1R) by two different small molecules, picropodophyllin and linsitinib, suffices to trigger autophagy in cancer cells, to augment the CD8/FOXP3 ratio in tumor-infiltrating lymphocytes, and to enhance therapeutic responses to chemotherapy and immunotherapy (8). Of note, this pathway appears to be clinically relevant. First, in breast cancer specimens, the activating phosphorylation of IGF1R determined by immunohistochemistry is associated with suppressed autophagy, a poor CD8/FOXP3 ratio, and dismal prognosis (8). Second, when patients with locally advanced breast cancer undergo so-called “fasting mimicking diet” (FMD) cycles (a hypocaloric regimen of 5 days covering approximately 40% of the normal nutrient uptake every 3–4 weeks) together with neoadjuvant chemotherapy, they manifest a reduction of free IGF1 in the plasma as well as signs of improved immunosurveillance, such as a contraction of circulating immunosuppressive myeloid and regulatory T cells as well as augmented intratumor cytotoxic T cell responses (9). Since anthracycline- and cyclophosphamide-elicited anticancer immune responses play a decisive role in tumor debulking (10), this may explain why FMD enhances the frequency of close-to-complete pathological responses in breast cancer patients treated with neoadjuvant chemotherapy (11). Although patient compliance is an issue in this type of study (9, 11), these results strongly suggest the clinical utility of fasting. Accordingly, multiple clinical assays have been launched to evaluate fasting regimens in the context of cancer immunotherapy. This applies to advanced skin malignancies (ClinicalTrials.gov NCT04387084), brain tumors (NCT02359565), head and neck cancer (NCT05083416), and non–small cell lung cancer (NCT03709147; NCT03178552; NCT03700437).

## Ketogenic diet

Fasting causes a reduction in glycemia coupled with an increase in so-called ketone bodies (mostly β-hydroxybutyrate), which are produced by the liver as a replacement fuel for the bioenergetic supply of vital organs, including the brain (12). Ketogenic diets, which are very low in carbohydrates, provide a strict minimum of protein and essentially consist of lipids; these diets induce a similar metabolic shift (glycemia with ketosis), including a reduction in circulating IGF1 levels (13). When mice are fed a ketogenic diet, they exhibit an improved T lymphocyte–dependent control of orthotopic skin melanomas and renal cancers (14). Moreover, a ketogenic diet enhances the efficacy of PD-1 blockade alone or together with anti–CTLA-4 antibodies. These effects are particularly strong when a ketogenic diet is administered in an intermittent rather than continuous fashion (14). Administration of sucrose in the drinking water breaks ketosis (meaning that β-hydroxybutyrate levels decrease to control levels) and abolishes the beneficial effects of the ketogenic diet on immunotherapy (14), evidence in favor of the hypothesis that ketone bodies are indeed required for immunostimulation (Figure 1B). Accordingly, the pharmacological antagonist of the β-hydroxybutyrate receptor GPR109A abolishes the antitumor effects of the ketogenic diet (14). More importantly, oral or parenteral administration of β-hydroxybutyrate is sufficient to sensitize tumor-bearing mice to immunotherapy with anti–PD-1 antibodies.

Mechanistically, β-hydroxybutyrate prevents the PD-1 blockade–induced upregulation of PD-L1 on myeloid cells, while enhancing the expansion of CXCR3^+^ T cells (14). Based on these preclinical results, a clinical trial (NCT05119010) has been designed to compare the effects of a ketogenic diet with those of orally administered β-hydroxybutyrate in renal cancer patients under immunotherapy with nivolumab (anti–PD-1) and ipilimumab (anti–CTLA-4). In this trial, the fecal microbiota of patients will be subjected to detailed analyses. Indeed, in mice, a ketogenic diet enhances the abundance of specific bacteria, such as *Eisenbergiella massiliensis*, which is also augmented in humans undergoing low-carbohydrate diet interventions and highly correlates with serum β-hydroxybutyrate concentrations (14). At this point, however, it has not been clarified whether *E*. *massiliensis* has immunostimulatory properties or whether this bacterium is a mere biomarker of compliance with the ketogenic diet. It will be important to measure the circulating concentrations of IGF1 and IGF1-binding proteins in patients enrolled in this trial to understand whether the depletion of circulating free IGF1 levels might contribute to immunostimulation by ketosis, similarly to what has been seen after fasting (Figure 1B).

## Supplementation

Beyond the gross interventions, such as fasting or ketogenic diet, discussed above, it is possible to perform more subtle dietary supplementations. For example, feeding mice bearing a variety of different tumors (breast cancer, fibrosarcoma, or melanoma) with the polyphenol-rich berry camu-camu (*Myrciaria dubia*) confers sensitization to PD-1 blockade, as it improves the CD8/FOXP3 ratio in the tumor immune infiltrate (15). A chemically defined polyphenol, castalagin, contained in camu-camu is sufficient to mediate these anti–PD-1–sensitizing effects as well as the increase in the CD8/FOXP3 ratio. Oral administration of the entire berry or castalagin induces shifts in the fecal microbiota, increasing the abundance of bacteria associated with efficient immunotherapeutic responses (*Ruminococcaceae* and *Alistipes*) (15). Of note, castalagin interacts with the cellular envelope of the *Ruminococcus* species, suggesting that it stimulates the expansion of this species through a direct trophic effect. In line with this hypothesis, feces from patients differed according to their capacity to digest castalagin to urolithin A, isourolithin A, and urolithin B, and metabolizers showed an increased abundance of *Ruminococcus bicirculans*, *Ruminococcus bromii*, *Alistipes finegoldii*, and *Alistipes onderdonki* (15). Thus, in two lung cancer patients receiving encapsulated camu-camu powder, fecal *R*. *bromii* expanded. Moreover, when germ-free mice with fibrosarcomas were gavaged with castalagin together with *R*. *bromii,* tumor progression was slowed down, and no such effect was found with either castalagin or *R*. *bromii* alone (15). Thus, the available evidence indicates that castalagin acts as a prebiotic, facilitating the proliferation of immunostimulatory bacteria. Of note, the castalagin metabolite urolithin A induces mitochondrial autophagy (mitophagy), suggesting that it may act as a postbiotic to improve human health (16). However, possible immunostimulatory effects of urolithins have not yet been reported (Figure 1C). A clinical trial (NCT05303493) will evaluate the capacity of camu-camu to amplify the immunotherapeutic response of patients with melanoma or non–small cell lung cancer to ICIs.

## Conclusions

Nutritional interventions are emerging as novel strategies for improving the outcome of treatments with PD-1/PD-L1–targeting ICIs. Mechanistically, such ICI-sensitizing regimens cause the induction of autophagy due to IGF1 depletion (as shown for fasting), enhance the levels of circulating β-hydroxybutyrate (as demonstrated for the ketogenic diet), or expand immunostimulatory bacteria (as exemplified for camu-camu). At this point, it remains elusive whether these mechanisms are exclusive for different dietary interventions or whether they intersect into a common pathway (Figure 1). Answering this question will open the possibility of replacing rough dietary interventions with the administration of molecular defined agents, including pharmacological IGF1 receptor inhibitors, the metabolite β-hydroxybutyrate, the prebiotic castalagin, and urolithin A, alone or in combination.

## Version 1 06/01/2022

Electronic publication
